# Smart-Responsive Nucleic Acid Nanoparticles (NANPs) with the Potential to Modulate Immune Behavior

**DOI:** 10.3390/nano9040611

**Published:** 2019-04-12

**Authors:** Morgan Chandler, Kirill A. Afonin

**Affiliations:** Nanoscale Science Program, Department of Chemistry, University of North Carolina at Charlotte, Charlotte, NC 28223, USA; mchand11@uncc.edu

**Keywords:** nucleic acid nanoparticles, NANPs, immunostimulation, dynamic, conditionally activated, RNA interference, RNA nanotechnology

## Abstract

Nucleic acids are programmable and biocompatible polymers that have beneficial uses in nanotechnology with broad applications in biosensing and therapeutics. In some cases, however, the development of the latter has been impeded by the unknown immunostimulatory properties of nucleic acid-based materials, as well as a lack of functional dynamicity due to stagnant structural design. Recent research advancements have explored these obstacles in tandem via the assembly of three-dimensional, planar, and fibrous cognate nucleic acid-based nanoparticles, called NANPs, for the conditional activation of embedded and otherwise quiescent functions. Furthermore, a library of the most representative NANPs was extensively analyzed in human peripheral blood mononuclear cells (PBMCs), and the links between the programmable architectural and physicochemical parameters of NANPs and their immunomodulatory properties have been established. This overview will cover the recent development of design principles that allow for fine-tuning of both the physicochemical and immunostimulatory properties of dynamic NANPs and discuss the potential impacts of these novel strategies.

## 1. Introduction

Nanotechnology has been integrated into many aspects of modern life [1] by providing a means of additional control over the unique physicochemical properties of functional moieties—including size, surface charge, and hydrophobicity, as well as their precise incorporation—and making them useful for biomedical applications. The ability to fine-tune these properties subsequently allows for the improved efficacy of therapeutic treatments and has implications for the future of personalized medicine [2,3,4,5]. Nucleic acids, including both DNA and RNA, represent a branch of biopolymers which additionally offer a biocompatible and programmable therapeutic approach. Beyond their traditionally known roles as passive carriers of genetic information, DNA and RNA have emerged as building materials for versatile biological drugs, called therapeutic nucleic acids (TNAs), which can take advantage of cellular pathways for the sensing, targeting, and silencing of a broad spectrum of various diseases, including asthma, cystic fibrosis, viral infections, and cancers [6,7]. TNAs are a diverse class of biomacromolecules that include antisense oligonucleotides, triplex-forming oligodeoxyribonucleotides, immunostimulatory oligos, catalytic oligos, inhibitory DNAs, interfering RNAs, and aptamers, which differ by composition, secondary structure, and mechanism of action [8]. Each TNA class may include multiple subtypes. For example, RNA interference (RNAi) inducers include siRNAs, miRNAs, and shRNAs, to name just a few. The great potential of RNAi technologies became apparent from the recent inspiring example of the very first siRNA therapeutic agent, patisiran (trade name ONPATTRO^®^), which was developed against polyneuropathy in patients with hereditary transthyretin-mediated amyloidosis and approved by the U.S. Food and Drug Administration (FDA) in 2018 [9], in addition to the many studies showing the versatility and modularity of TNAs [10,11,12]. Usually, TNAs affect the flow of genetic information, mimic antibodies, or stimulate the immune system, causing either the suppression of disease-specific genes or the stimulation of gene expression in response to an antigen. Besides specific siRNAs, several other promising therapeutically potent classes of nucleic acids, such as antisense oligonucleotides, aptamers, DNA decoys, and ribozymes, are also under consideration [13]. Currently, three additional successful examples of TNAs are approved for therapeutic uses: fomivirsen (brand name Vitravene™), an antisense oligonucleotide designed against cytomegalovirus retinitis which was also the very first nucleic acid-based therapy approved by the FDA [14]; mipomersen (trade name KYNAMRO^®^), an antisense oligonucleotide approved for the treatment of homozygous familial hypercholesterolemia [15]; and pegaptanib (brand name MACUGEN^®^), an aptamer approved for the therapy of age-related macular degeneration [16].

If several different TNAs are simultaneously chosen for the same treatment and they must target the same cells, the optimal route for their controlled co-administration would be through the introduction of nucleic acid-based nanoparticles, or NANPs [17,18,19,20,21,22,23,24,25,26,27]. This strategy has resulted in multiple new methods for the design and assembly of nano-TNAs, in which nucleic acids also serve as building blocks to assemble programmable scaffolds with well-defined properties. To achieve this, biomedical sciences benefit not only from the natural roles of nucleic acids, but also from their known ability to form both canonical Watson–Crick (e.g., G–C and A–U (or T for DNA)) and non-canonical base pairings [28]. Non-canonical base pairs are mostly characteristic of RNAs and include 12 basic geometric families [28], thus leading to a diverse set of oligonucleotide structures, called motifs, that can fold into complexes with a precise 3D shape. The existing RNA motifs [25,29,30,31,32,33,34,35] can be rationally combined to promote the assembly of various NANPs that can be further decorated with therapeutic domains and employed as drug delivery platforms [36]. Such a novel use of RNA as a starting material in bottom-up assembly has helped to establish the burgeoning field of RNA nanotechnology, which also utilizes the biological functions of nucleic acids to address specific biomedical challenges [21,33,36]. Programmable NANPs guarantee precise control over versatile functionalization with different moieties, such as aptamers, fluorescent dyes, and proteins, and their simultaneous delivery with numerous siRNAs targeting different biological pathways [20,21,22,23,25,31,37]. NANPs’ ability to successfully combat diseases at their source has already been confirmed by multiple animal studies [25,38,39,40,41,42,43]. Additionally, the design principles being developed in RNA nanotechnology address fundamental problems relevant to the biophysics of RNA co-transcriptional folding [24,44,45,46,47], structure–activity relationships [48], and their physicochemical interactions with other classes of biomolecules (e.g., lipids [41,49,50], proteins [51,52]) or inorganic materials [53,54].

**Figure 1:**
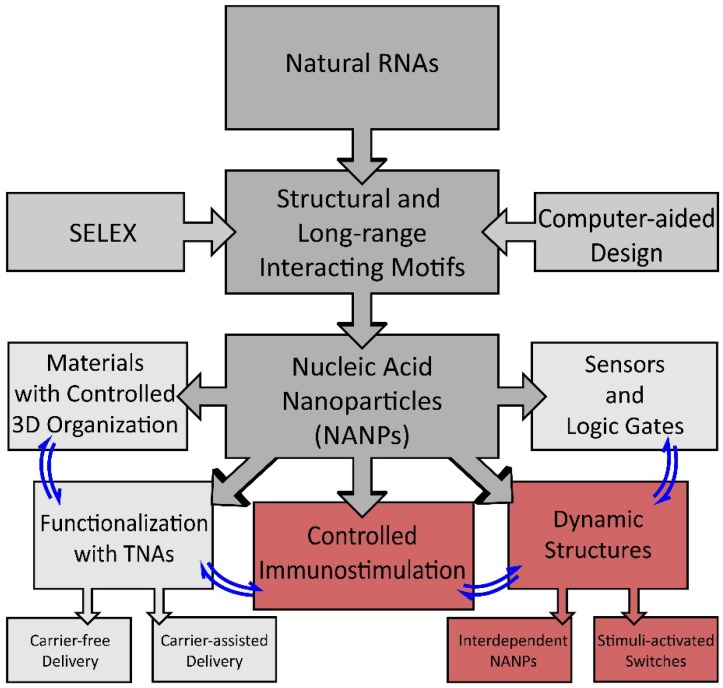
Schematic of the development of nucleic acid-based nanoparticle (NANP) technologies and their current applications. Structural motifs inspired by structure–activity studies of natural RNAs, computer-assisted design, or determined by SELEX (systematic evolution of ligands by exponential enrichment) are used for the rational design of NANPs. NANPs can then be programmed for multiple applications for materials organization, sensors, and dynamic structures, or can be functionalized with therapeutic nucleic acids (TNAs) or used to elicit a controlled immune response.

Though TNAs in general and NANP-based nano-TNAs in particular are strong candidates in nanotherapeutics, their transition into a clinical setting has been hindered by a lack of general knowledge about their immunostimulatory properties [55], while their statically designed structures have posed limits to the conditional activation or deactivation of preprogrammed biological functions. To overcome these obstacles, recent efforts, as described in this review, have been focused on a new platform of design principles for assembling dynamic nucleic acid assemblies with a controlled and fine-tunable immune response (Figure 1).

## 2. Dynamic Shape-Switching and Functional Activation with NANPs

**Figure 2:**
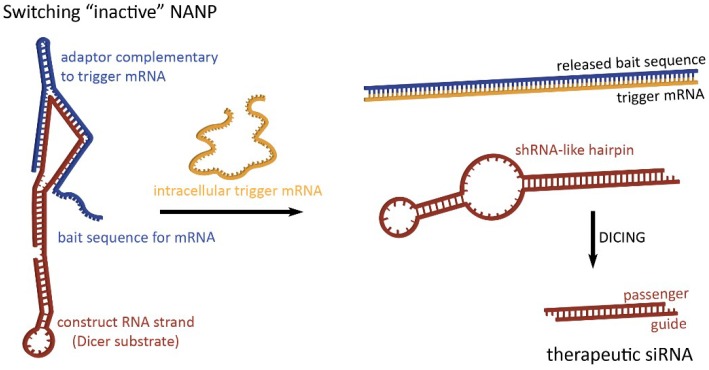
Dynamic NANPs exemplified by two-stranded RNA switch. The presence of trigger mRNA promotes strand rearrangements in an otherwise inactive switch, thus allowing for the release of therapeutic siRNA.

To take full advantage of the programmability of nucleic acids, therapeutic NANPs could be conditionally activated inside human cells for the release of preprogrammed functionalities which would offer higher targeting specificity, thereby potentially reducing off-target effects. The “dynamicity” preprogrammed in NANPs’ behavior defines their ability to be activated in response to various stimuli. By interacting with, for example, a target strand or environmental variable of choice as a diagnostic step, switching NANPs can be designed to release therapeutics only when these interactions occur [56,57]. In the absence of the predetermined intracellular trigger mRNA, characteristic only for the diseased cells, these switching NANPs are not in active therapeutic conformations (Figure 2). The single-stranded bait sequences made of RNA (or chemically modified nucleotides) that deactivate switching NANPs are computationally designed to provide a thermodynamically more favorable binding to the trigger mRNA than to NANP strands [56,57]. The interactions between the switching NANPs and trigger mRNA removes the bait strand and consequently exposes shRNA-like hairpins that become the next most thermodynamically stable fold of the remaining NANP. The human enzyme Dicer can now recognize and cleave these refolded structures and load the RISC with the “guide” strand which, in turn, will activate RNAi. RNAi activation results in the suppression of targeted anti-apoptotic genes, thus inducing the programmed death of the diseased cell.

Besides activation from environmental stimuli, RNA/DNA assemblies can also be designed to interact with their cognate counterparts for their activation [40]. Hybrid structures composed of both RNA and DNA (or chemical analogs [58]) can be used to synchronize the activation of functionalities embedded into the hybrid structures [40,59]. Thus, for RNAi activation, two halves of a Dicer substrate (DS) RNA [60] can be split between two hybrid duplexes which undergo strand displacement when both are present to form a complete duplex for processing and subsequent gene silencing. The process of reassociation is thermodynamically driven and can be initiated via complementary ssDNA [40,61,62] or ssRNA [26] toehold interactions. The use of hybrids also offers some preclinical benefits, such as controlled rates of reassociation, significantly reduced degradation in human blood serum, and the possibility to chemically introduce additional functionalities into the DNA strands without affecting the function of the released RNAs. Besides RNAi inducers, split aptamers have also been tested which, when presented as separate halves, are non-functional. However, when the halves of the split aptamer are brought together during the reassociation, the completed aptamer regains its function [40,63,64]. The development of split fluorescent aptamers, such as malachite green [65], Spinach [63], and Broccoli [66], has produced tools which are especially useful when applied as a validating output for dynamic RNA nanotechnology [63,64,66,67,68], in the visualization of NANP assemblies [45,69], and for logic gating [70].

**Figure 3:**
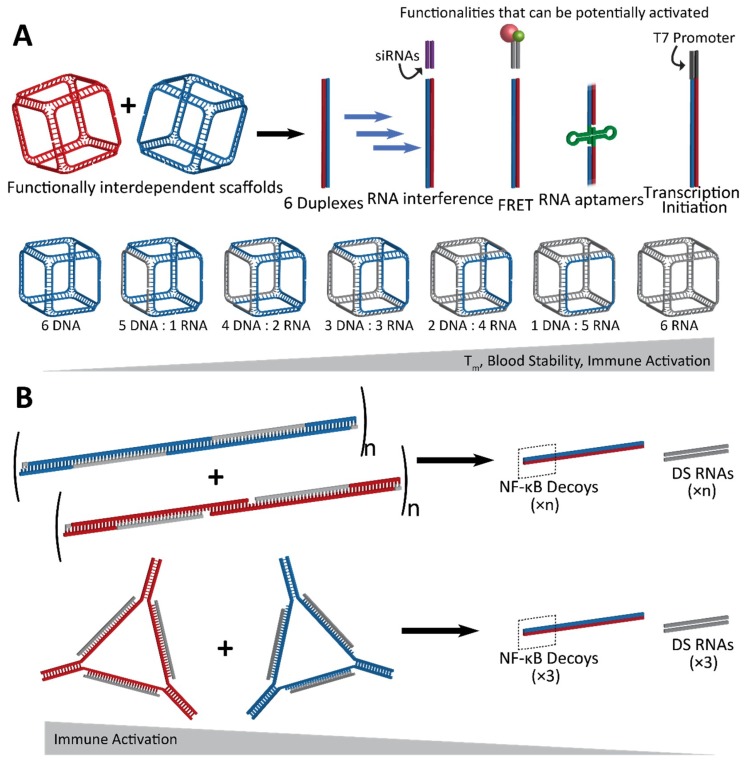
Conceptual representation of interdependent shape-switching NANPs programmed to activate multiple functionalities upon their isothermal reassociation. (**A**) Hexameric NANP cubes and complementary anti-cubes reassociate to drive the formation of six double-stranded duplexes. Functionalities which can potentially be added to the NANPs are split between the two functionally interdependent cubes to become active upon reassociation, resulting in the completion of siRNAs for gene silencing, Förster resonance energy transfer (FRET) pairs for visualization, aptamers, or T7 RNA polymerase promoter sequences for the co-transcriptional assembly of RNA NANPs. The cubes can be composed of varying ratios of DNA and/or RNA, with melting temperature, blood stability, and immune activation increasing with RNA composition. (**B**) Hybrid DNA/RNA fibers (top) and polygons (bottom) reassociate to form DNA duplexes containing NF-κB decoys, as well as Dicer substrate (DS) RNA duplexes for gene silencing. The NF-κB decoy binds to and prevents NF-κB from entering the nucleus, thereby stopping it from producing pro-inflammatory cytokines. As a result, immune activation decreases as the hybrid structures reassociate.

Another design scheme, which evolved from the approach using RNA/DNA hybrids, utilizes NANPs which are designed to completely reassociate with one another and thus activate embedded functionalities. An example of such conditional activation has been shown via interdependent NANPs in the shape of three-dimensional cubes which interact with their cognate “anti-cubes” under isothermal conditions to trigger a change in the shape and successive activation of attached functionalities [71]. Cubes are composed of six strands of RNA and/or DNA, while the anti-cube strands are designed as the reverse complements of the cube sequences (Figure 3A). Only two NANPs—the cube and anti-cube—are required for activation. Upon interaction, the two cubes that each have a total of nine unpaired bases per corner undergo a thermodynamically driven switch into six double-stranded duplexes. Moieties which are split across the cube and anti-cube complement strands are then brought together for activation of functionality.

### 2.1. Activation of RNA Interference, FRET, RNA Aptamers, and Transcription Initiation

Conditional activation of the functionalities upon reassociation of the cube and anti-cube has been demonstrated with a variety of methods which also establish the wide range of applications for this technology [71]. A set of cognate cubes were decorated with split DS RNAs against multiple different genes (*BCL2, PLK1,* and green fluroscent proteins—*GFP*). DS RNAs were labeled with fluorophores chosen to undergo a Förster resonance energy transfer (FRET) for real-time intracellular analysis. Upon reassociation, the formation of DS RNA duplexes could be further processed in a cellular environment, through dicing, for the release of functional siRNAs. Cognate functionally interdependent NANPs carrying DS RNAs against GFP [60] showed a significant knockdown in the fluorescence of human breast cancer cells expressing enhanced GFP [71]. Also, the downregulation of *BCL2* and *PLK1* genes [27], which have been shown to induce apoptosis, was confirmed by a significant decrease in the viability of human cervical and prostate cancer cells.

The reassociation of the cube and anti-cube and the consecutive formation of RNA fibers was also demonstrated with the activation of a split aptamer. The RNA aptamer Broccoli [72], which binds the chemically synthesized fluorophore DFHBI-1T to mimic the natural function of GFP, was split into two separate strands termed Broc and Coli [66,68]. With both parts of the aptamer attached to cognate RNA cube strands, the Broc cube and Coli anti-cube reassociated in the presence of DFHBI-1T, allowing for the interaction to be traced via fluorescence in real time [71].

The reassociation of the DNA cubes and anti-cubes carrying split T7 RNA polymerase promoters was shown to form templates for the co-transcriptional assembly of complete RNA cubes in vitro. Anti-cubes carrying a complete T7 promoter can be used for the RNA cube’s co-transcriptional assembly, thus providing a template for the future intracellular production of RNA nanoparticles that can be activated upon interaction of functionally interdependent NANPs [71].

Lastly, hybrid RNA/DNA fibers and polygons can interact with their cognate structures for reassociation, resulting in the release of DS RNAs as well as double-stranded DNAs carrying NF-κB decoys (Figure 3B) [73]. NF-κB (nuclear factor kappa-light-chain-enhancer of activated B cells) is expressed in most animal cells and generally remains in an inactive state in the cytoplasm until it can be activated for translocation to the nucleus, where it is then involved in the production of pro-inflammatory cytokines. With the release of NF-κB decoys [74,75], which can bind and retain NF-κB in the cytoplasm, the hybrid fibers and polygons dynamically modulate the immune response in addition to RNAi activation. Further, this strategy takes advantage of all strands included in the assembly to utilize them in functional roles, leaving no static byproducts [73].

### 2.2. Fine-Tunable Properties

Besides the activation of multiple functionalities, an important feature of the rational design of the cube/anti-cube NANP system is the ability to fine-tune the physicochemical and immunological properties of NANPs simply by adjusting the ratios of DNA and RNA strands in their composition [71]. With an increasing number of RNA strands, both the thermodynamic stability and the reassociation time of cubes increased. The immune response to these NANPs was also assessed via the measurement of the activation and secretion of interferon (IFN)α and a panel of pro-inflammatory cytokines and chemokines (IL-1β, TNFα, IL-8, and MIP-1α). NANPs composed of all RNA strands were the most potent stimulators of an immune response, indicating that they may serve an application as a vaccine adjuvant. By adjusting the nucleic acid composition, the immune response to nanoparticles can be fine-tuned in such a way that assemblies could be utilized for drug delivery or immunotherapy.

## 3. Immunostimulatory Properties of NANPs

**Figure 4:**
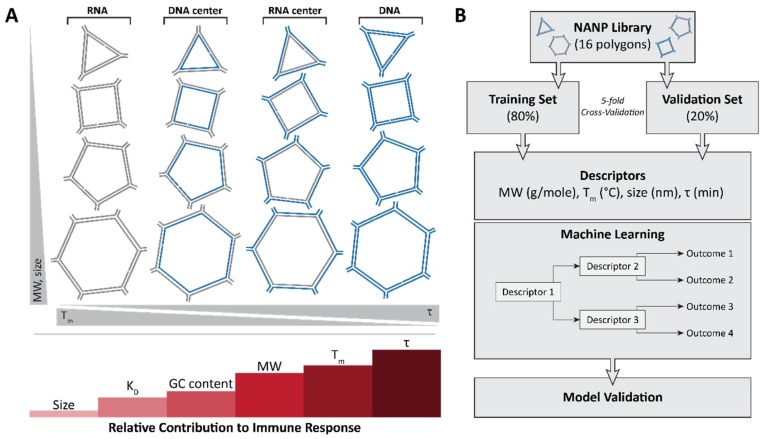
The very first application of quantitative structure–activity relationship (QSAR) modeling for NANP immunostimulation uses measured physical and chemical properties as inputs to predict a given NANP’s ability to generate a pro-inflammatory response. (**A**) A library of 16 NANPs which incorporate the same sequences was used to determine the physicochemical properties contributing to immune response. From the study, size (diameter) was determined to contribute the least to immune response, followed by K_D_, GC content, molecular weight, T_m_ and, finally, stability with the highest contribution into the random forest model. (**B**) A schematic showing the QSAR modeling approach which uses the 16 NANP library.

It is becoming apparent that interactions between NANPs and the immune system must be defined to permit the successful translation of this technology to the clinic. Foreign nucleic acids can produce a robust and severe response in immune cells. Pro-inflammatory cytokines and type I interferons are characteristic of nucleic acid sensing in immune cells, and in animals, it can produce anywhere from minor inflammation to severe cytokine storms. Therefore, to address fundamental questions regarding the immune recognition of these novel materials in a timely fashion, the relation of features such as the size, shape, composition, and physicochemical properties of various polygonal NANPs [76] to the activation of immune responses in human microglia-like cells (hµglia or hHµ) was examined [48] using a series of assembled RNA, DNA, and RNA/DNA hybrid NANPs. A set of several polygons designed based on the versatile tetra-U helix linking motif [76] was assembled by using a ubiquitous set of strands (both RNA and DNA) so that their immunostimulatory effects—when composed of all RNA, RNA with a DNA center, all DNA, and DNA with a RNA center—could be characterized (Figure 4A). The engineered NANPs were designed to assume various shapes and sizes as well as variations in their content (RNA vs. DNA) and physicochemical stabilities while having minimal differences in their sequences. The measured biomarkers of a pro-inflammatory response were cytokines and type I interferons. Using modern machine learning techniques, the quantitative structure–activity relationship (QSAR) models were developed to successfully predict and engineer NANPs able to stimulate an intended immune response, or lack thereof [48].

This very first application of QSAR modeling for NANPS studied measured physical and chemical properties as inputs to predict a given NANP’s ability to generate a pro-inflammatory response (Figure 4B). Importantly, this QSAR model [48] can be used to more intelligently design nucleic acid-based pharmaceuticals to reduce detrimental immune responses, stimulate desired protective immune responses, and increase their intended activity. This work is instrumental in bridging the rapidly narrowing gap between basic research on NANPs and advanced pharmaceuticals containing these novel materials.

**Figure 5:**
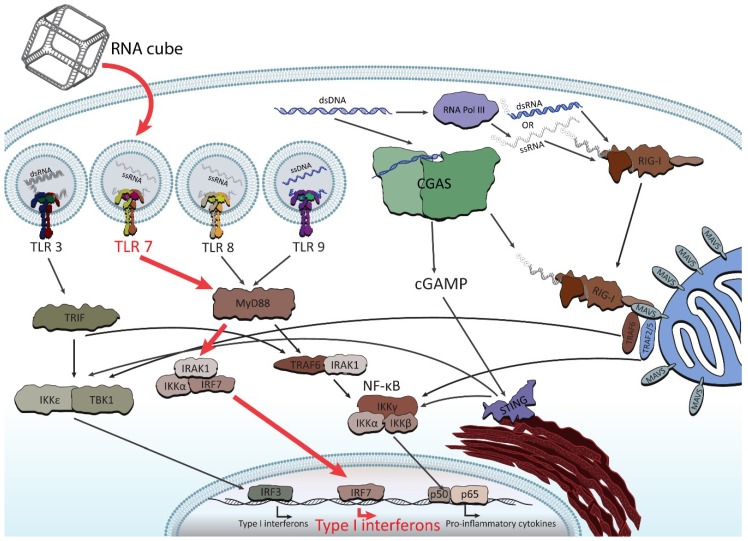
Abridged illustration detailing the pathways involved in the endosomal and cytosolic sensing of smaller therapeutically relevant nucleic acids. NANPs have been observed to enter through the endosomal pathway (when complexed with Lipofectamine 2000) and trigger the toll-like receptors (TLRs). The pathway shown in red (for TLR 7) demonstrates the identified route for RNA cube recognition. For the purpose of this review, the figure shows all individual TLRs (related to NA sensing) in separate endosomes in order to better highlight the particular pathway of NANPs’ recognition.

Following these findings, the very first systematic investigation of NANP recognition by immune cells using primary human peripheral blood mononuclear cells (PBMCs) from a cohort of more than 100 healthy human donors was recently designed and executed [77]. Despite expectations [78], the researchers did not find a strong, uniform immune response for all NANPs. Instead, the tests found varying and specific responses from different immune cells, depending on each NANP’s shape and formulation. It was discovered that all NANPs used without a delivery carrier were immunoquiescent, and that type I and III IFNs are key cytokines triggered by NANPs after their internalization by phagocytic cells. It was shown that overall immunostimulation relies on the NANPs’ shape, type of connectivity, and composition. Importantly, plasmacytoid dendritic cells were identified as the primary interferon producers among all PBMCs treated with NANPs, and it was demonstrated that scavenger receptor-mediated uptake and endosomal toll-like receptor (TLR) signaling are essential for NANPs’ recognition. In particular, TLR 7 (Figure 5) was identified as a key player in immunostimulation triggered by NANPs that was observed both in model HEK-Blue TLR 7 cells [77] and in human PBMCs [79], as well as being later confirmed by extensive mechanistic studies [79]. All immunological studies strongly suggest that the further understanding of how particular NANPs can trigger the immune response is required to open up possibilities in a new field where NANPs can be used as vaccine adjuvants.

**Figure 6:**
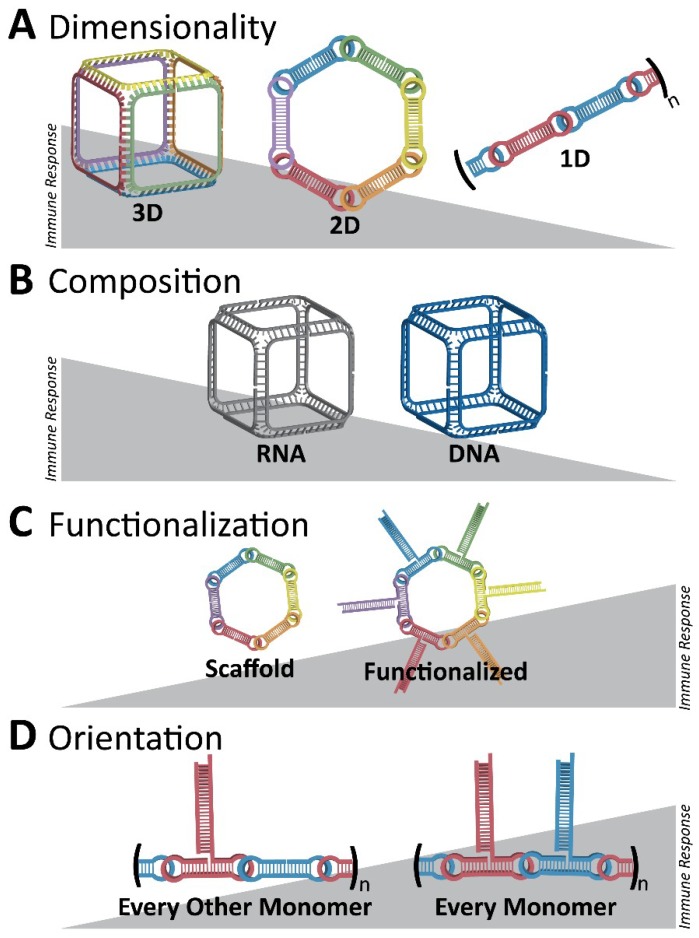
Initial overall immunostimulatory trends observed with variations in NANP designs. (**A**) Three-dimensional RNA cubes are potent immunostimulants, followed by two-dimensional RNA rings and one-dimensional RNA fibers. (**B**) Between structures of the same dimensionality, RNA NANPs are more immunostimulatory than DNA NANPs. (**C**) A NANP scaffold becomes more immunostimulatory if it is functionalized, for example, with TNAs. (**D**) For RNA fibers which are less immunostimulatory in terms of dimensionality, functionalization at every monomer results in a greater immune response than if the fibers are functionalized at every other monomer. Different colors emphasize the architecture of NANPs consisting of multiple strands (A,C,D) or changes in their composition (B) such as DNA vs. RNA.

Importantly, the following work has also demonstrated that the functionalization of NANPs with RNAi inducers completely changes their known immunorecognition [80]. However, the possibility to control the magnitude and specificity of the immunostimulatory response by varying the design parameters and functional moieties in each NANP has also been revealed (Figure 6). Additionally, through using RNA fibrous structures which have been shown to have minimal recognition by the immune system, the delivery of multiple modalities, such as siRNAs and fluorophores, can be coordinated with minimal immunorecognition. Relying on HIV-like (~180°) kissing loop interactions, dumbbell-shaped hairpins are the modular building blocks for these assemblies and allow for simple customizability [80].

## 4. Conclusions

In conclusion, the responsive behaviors of NANPs can be determined by the specific design principles for individual NANPs, their assembly type, compositions, physicochemical properties, and the presence of any additional functionalities in their structure. The use of NANPs for the controlled design of dynamic stimuli-responsive systems presents multiple advantages: (i) NANPs can be programmed to gather multiple different functionalities for their simultaneous delivery to cells [17,25,37,42,61]; (ii) RNA NANPs and their chemical analogs can be co-transcriptionally assembled [23,24,45,46]; (iii) the relatively inexpensive production of NANPs with a high batch-to-batch consistency [22] enables their economic industrial scale production; (iv) the thermal and chemical stabilities of NANPs can be fine-tuned by introducing chemically modified nucleotides or DNA strands into their composition [45,71,77]; (v) carrier-free NANPs avoid nonspecific cell penetration due to their negative charge [77] and, thus, can be used for extracellular tasks; and (vi) the immunological properties of NANPs are controllable and can potentially be predicted [48,77]. The ability to fine-tune immunostimulation and the conditional activation of functionalities are important facets in the rational design strategies of programmable biopolymers. Stimulation of the controlled production of IFNs and pro-inflammatory cytokines by human immune cells is instrumental for immunotherapies and vaccine use when activation of the immune response is necessary [77]. The excessive and uncontrolled induction of cytokines, however, may promote tissue necrosis and become harmful [81]. The innovative development of NANP-based tools that can be used for communication with the human immune system can be employed in achieving the desirable activation of the immune system for vaccines and cancer immunotherapies, while immunoquiescent NANPs can be used as nano-TNAs and for dynamic NANP construction.

Predicting and controlling the immune responses triggered by NANPs, especially when the magnitudes of those responses may vary between donors, remains a substantial challenge for the furtherment of nano-TNAs. In addition, due to their negative charges which inhibit free passage across the cell membrane, a carrier or targeting moieties are needed for delivery. While there is a plethora of nanoparticles formulated for nucleic acid delivery, the effects of the carrier on the immunostimulatory properties of NANPs remain unclear. Furthermore, the healthy host immune tolerance to nucleic acids and the generation of antibodies against DNA and RNA after exposure to NANPs is an important safety topic which, so far, has not been addressed. Thus, assessing the immunogenicity of NANPs is a future logical direction in this field.

The activation of RNA interference, FRET, RNA aptamers, transcription, and other responses upon the reassociation of cognate NANPs at isothermal conditions exhibits the potential for dynamic constructs to operate only in the presence of a target. Additionally, the degree to which the properties of TNAs contribute to the elicitation of an immune response will allow for their intentional activation or evasion, depending on whether immunotherapy or drug delivery is desired. By optimizing conditional activation and immunostimulation, NANPs can be better designed for future clinical applications.
